# Hans Joachim Scherer and His Impact on the Diagnostic, Clinical, and Modern Research Aspects of Glial Tumors

**DOI:** 10.7759/cureus.6148

**Published:** 2019-11-13

**Authors:** George S Stoyanov, Lilyana Petkova, Deyan L Dzhenkov

**Affiliations:** 1 General and Clinical Pathology, Forensic Medicine and Deontology, Medical University of Varna, Varna, BGR

**Keywords:** glioblastoma multiforme, scherer figures, growth pattern, diagnostic criteria, invasive growth

## Abstract

The historical descriptions of glial tumors are often poorly understood and interpreted. The gross and histological depictions of glial tumors are often credited to Virchow, and while the first true histological description is truly his, gross descriptions can be traced back to the beginning of the 1800s, with their classification and histogenesis attributed to Percival Bailey and Harvey Cushing. Without any question, the most prominent and under-credited researcher in the field of glioma pathobiology was the German neuropathologist Hans Joachim Scherer. Despite the limited armamentarium available to him, his systematic approach led to conclusions, some of which have now been molecularly explained today while some are still being widely researched. Scherer defined pseudopalisadic necrosis as a pathognomonic feature of glioblastoma multiforme (GBM), as well as secondary features due to tumor growth, known collectively as secondary Scherer figures, for example, neuronal and vascular satellitosis, tract and subpial aggregation. All these features are key points in the modern histological diagnosis of glial tumors. Other contributions by Scherer include the definition of glomeruloid vascular proliferation and his conclusion that they are caused by vascular factors released by the tumor, decades before vascular endothelial growth factor and its receptors were discovered and their role in glioma evolution was established. Furthermore, he concluded that GBMs can arise de novo (primary) or from a preceding lower-grade glioma (secondary). All his contributions find their place in all modern aspects of glioma research, with some giving a simple explanation of the phenomena observed in glial tumors.

## Introduction and background

The history of glial tumors and the major contributors to the establishment of their concepts are often misunderstood and interpreted. Scientists like Rudolph Virchow, Percival Bailey, and Harvey Cushing are often credited and praised for accomplishments that were not theirs, whereas many other authors are often overlooked and forgotten. This lack of scientific continuity has often led to a rediscovery of already established phenomena and swaying away from promising fields of research in favor of others, yielding little to no results.

## Review

The gross and histological depiction of glial tumors are often credited to the German pathologist Rudolph Virchow (born on October 13, 1821, and died on September 5, 1902), and while the first true histological description is truly his, gross descriptions can be traced back to the beginning of the 1800s in the English, French, and German scientific literature [1-4].

Another case in point is the coining of the histological criteria on which the modern diagnostic guidelines are based. Almost always they are credited to the American neuropathologist Percival Bailey (born on May 9, 1892, and died on August 10, 1973) and prolific neurosurgeon Harvey Cushing (born on April 8, 1869, and died on October 7, 1939), and while their classification is the basis of the modern World Health Organization classification of tumors of the central nervous system (CNS), it was aimed at purely comparing the tumor conglomerates with the healthy cells of the CNS and underwent several revisions by the authors themselves [3,5-6].

**Figure 1:**
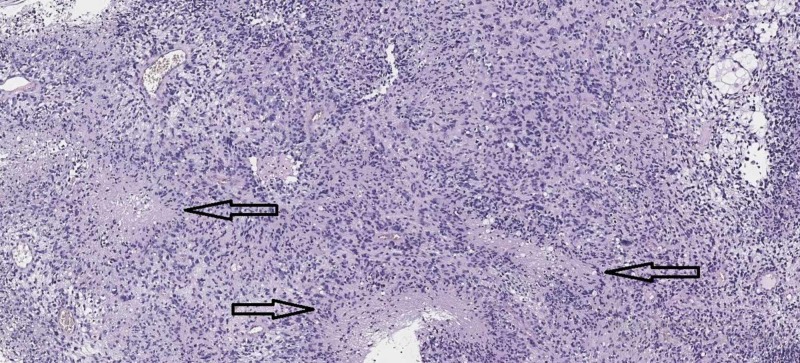
Scherer figures – pseudopalisadic necroses (arrows) Hematoxylin and eosin staining (original magnification 50x)

Without any question, the most prominent and under-credited researcher in the field of glioma pathobiology was the German neuropathologist Hans Joachim Scherer (born on May 14, 1906, in Bromberg, Kingdom of Prussia [now Bydgoszcz, Poland], and died on April 16, 1945, in Landshut, USSR-occupied Germany) who made some of the most important observations and conducted research on glial tumors, especially glioblastoma multiforme (GBM), such as the pathognomonic pseudopalisadic necrosis, sometimes referred to as Scherer structures in his honor (Figure 1) [7-9]. These tumor necroses have a specific cellular arrangement around them (pseudopalisadic) due to the tumor cells in GBM trying to escape from the ischemic area to the more richly supplied areas of the tumor.

In 1933, Scherer published his first article on glial tumors, focusing primarily on GBM and emphasizing the importance of mesenchymal components in GBM, such as mesenchymal-like stroma, mesenchymal proliferation in regressive tumor changes, and formation of glomeruloid vascular proliferation in such tumors [10-11]. In terms of the glomerular structures, Scherer postulated that it may be a reactive process or a part of the development of neoplasia itself.

In his subsequent studies, Scherer postulated that vascular factors were a result of the development and progression of GBM; hence, the tumor would lead to the formation of the vessels. He postulated that the presence of vascular factors led to the formation of such vessels, most likely as a result of necrotic tumor cells in poorly vascularized areas, as he also noticed the specific spatial arrangement of the formations [9]. These conclusions were made decades before the discovery of vascular endothelial growth factor and its receptors and today have an established role in the development of GBM and neoplastic vascularization [12].

In 1940, Scherer made a dictum on the basis of his observations, which surpassed other findings on the subject for decades, postulating the presence of both primary and secondary GBMs on the basis of the clinical course: primary GBMs develop rapidly, without a previous lesion (de novo), whereas the secondary ones arise on the background of previously reported lower-grade glioma and have a slower clinical evolution [4].

Scherer’s impact on glioma research can even be traced to the most minor and rarely observed features of GBM, as he studied the tumors extensively, not only on biopsy material but also on autopsy brains, where he studied the grossly healthy parts for possible tumor micro-involvement [9-11].

Thus, he described foci of tumor cells in distant parts of the brain, postulating that radical surgical excision was impossible and explaining the high and fast recurrence rates, not only in GBM but also in lower grade glial tumors, at a time when hemispherectomies were still being practiced and recurrences were observed in the contralateral hemisphere as early as six months postoperatively [11].

Tumor growth was also an important area studied by Scherer, as again on autopsy material he described the phenomena of subpial and tractal aggregation, and perineuronal and perivascular satellitosis (tumors cells distant from the main tumors mass surrounding and aggregating along with the healthy structures of the brain parenchyma) [12]. These phenomena not only explain the diffuse growth of glial tumors but also explain why they present clinically at a late stage, the reason being the long-time period needed for the neurons and other structures to be destroyed by the neoplastic cells, unlike in metastatic disease.

**Figure 2:**
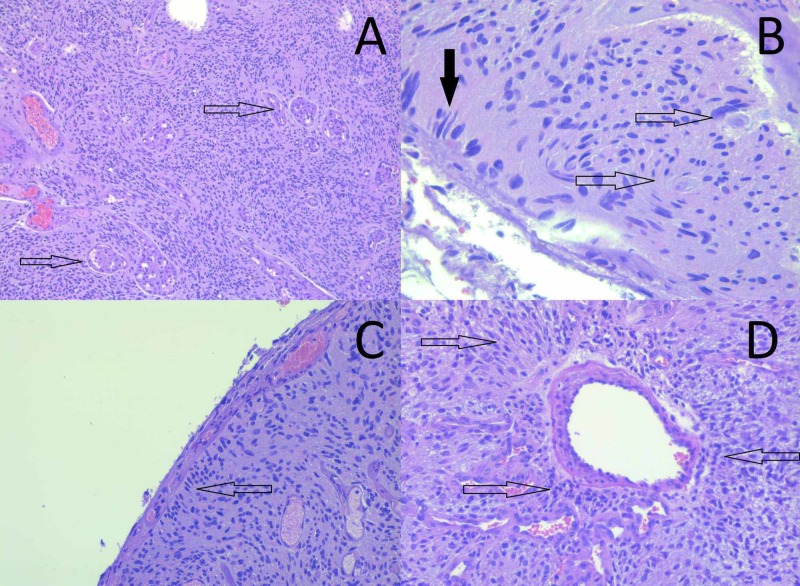
Secondary Scherer figures (A) Glomeruloid vascular proliferation (arrow) (hematoxylin and eosin staining, original magnification 100x). (B) Neuronal satellitosis (hollow arrow) and subpial palisading of tumor cells (black arrow) (hematoxylin and eosin staining, original magnification 400x). (C) Subpial palisading of tumor cells (arrow) (hematoxylin and eosin staining, original magnification 200x). (D) Perivascular satellitosis of tumor cells (arrow) (hematoxylin and eosin staining, original magnification 400x).

Rarely observed on biopsy material, these phenomena of invasive but non-destructive growth are collectively known as secondary Scherer figures, not as pathognomonic as the primary features for GBM but indicative of glial tumors and not metastatic (Figure 2) [12].

All these observations by Scherer are now key points in the diagnostic process and clinical management of patients with glial tumors, yet they are often attributed to other authors.

All of Scherer’s work gives a basic explanation of not only the histological diagnosis of GBM but also several clinically observed phenomena and some modern aspects of glioma research. Firstly, GBM is one of the few malignant tumors, despite its aggressive nature, that does not give rise to a metastatic disease [13]. The classic example of cerebrospinal fluid metastasis to other parts of the CNS, although rarely observed, is always a late phenomenon, where the meninges or ependymas disrupt, allowing the neoplastic cells to migrate through the cerebrospinal pathways [14-16]. Most commonly, this phenomenon in the 21st century is attributed to surgical intervention, leading to iatrogenic destruction of these structures but not disease progression. Secondly, the few described lymph node metastasis cases are most often attributed to the placement of a ventriculoperitoneal shunt [17].

All these unique clinical features of GBM can be explained by the histological findings. GBM is a highly malignant and aggressive tumor that grows diffusely through the physiological structures of the CNS without destroying them [12-13].

Furthermore, the patterns of growth show a specific growth pattern in the CNS. The possibility of a growth factor or other substance found only in the CNS needed for the proliferation of the neoplastic cells and not found in other organs is one possible explanation. Therefore, despite the presence of circulating tumor cells in non-surgically treated patients, and their increased number after surgical treatment, these cells find it extremely difficult to initiate invasive growth into other structures [12].

The dense vasculature of GBM, including glomeruloid vascular proliferation and primary Scherer figures, would allow for many tumor cells to be detached from the main tumor mass and enter damaged blood vessels [18]. Tumor cell remnants, such as exosomes in GBM, are found in the systemic circulation, and their quantity is comparable to exosomes found in other cancer types, further supporting the theory that GBM has access to metastatic pathways; however, its unique pattern of growth inhibits the evolution of metastatic disease [18-19].

## Conclusions

Hans Joachim Scherer provided not only the basics but also the in-depth histological criteria and definitions of glial tumors, especially GBM, with important clinical correlations, despite the limited armamentarium available to him. Sadly, the geopolitical circumstances and Scherer’s Jewish heritage restricted much of his research, and his untimely death at the end of World War II led his name to be forgotten and his contributions being attributed to other authors. Despite the limitations to his research career, Scherer made innovative and bold statements on the topic, all of which are still true to this day and have found their morphological, clinical, and molecular basis. He reached these conclusions based on the observations of tumors, remaining brain parenchyma and the overall clinical history of the patients. His systematic approach allowed him to escape from the purely morphological aspects of pathology and to be one of the first to define prognostic factors, long before this became a tendency in modern medicine.
